# Safety Evaluation of a Medical Congress Held During the COVID-19 Pandemic—A Prospective Cohort

**DOI:** 10.3389/ijph.2022.1604147

**Published:** 2022-02-16

**Authors:** Johannes Sumer, Domenica Flury, Christian R. Kahlert, Nicolas J. Mueller, Lorenz Risch, Susanne Nigg, Marco Seneghini, Pietro Vernazza, Matthias Schlegel, Philipp Kohler

**Affiliations:** ^1^ Division of Infectious Diseases and Hospital Epidemiology, Cantonal Hospital St. Gallen, St Gallen, Switzerland; ^2^ Division of Infectious Diseases and Hospital Epidemiology, University Hospital Zürich, Zürich, Switzerland; ^3^ Labormedizinische Zentrum Dr Risch, Bern, Switzerland

**Keywords:** COVID-19, safety concept, congress, hygiene measures, capillary, dried-blood spots, serology, prospective cohort

## Abstract

**Objectives:** During the COVID-19 pandemic, few scientific congresses have been held on-site. We prospectively evaluated the safety concept of the congress of the Swiss Societies of Infectious Diseases and Hospital Hygiene.

**Methods:** The congress was held in Geneva (Switzerland) while local COVID-19 incidence (with SARS-CoV-2 wild type circulating) was 65/100,000 population (September 2020). A rigorous safety concept was implemented. Congress attendees filled out a questionnaire to assess risk perception, exposures, symptoms and diagnoses of SARS-CoV-2 before, during and after the congress. Dried blood spots were taken on-site and 4 weeks later to detect SARS-CoV-2 seroconversions.

**Results:** Of 365 congress attendees, 196 (54%) either answered the questionnaire (N = 150) or provided baseline and follow-up blood samples (N = 168). None of the participants reported a positive PCR in the 2 weeks after the congress. Five of 168 (3%) participants were seropositive at follow-up, all of which had already been positive at baseline.

**Conclusion:** Findings indicate that congresses with a rigorous safety concept may take place, even in areas with moderately-high COVID-19 activity. Whether this holds true in vaccinated populations and with more transmissible viral variants circulating remains unclear.

## Introduction

An important and widely recommended measure to reduce the spread of Severe Acute Respiratory Coronavirus-2 (SARS-CoV-2) is to ban or to limit public gatherings [1]. On November 17th, 2020, the Center for disease Control and Prevention (CDC) issued recommendations for organizers regarding risk assessment and safety concepts of such gathering [2, 3]. Medical congresses are also subject to these regulations; as a consequence, many medical and non-medical congresses were cancelled or converted into purely online events with different sensations about their output [4–7]. At the same time, scientific exchange, inspiring discussions with colleagues, and networking are more important than ever, in particular for infectious disease and infection prevention specialists.

After the first COVID-19 wave in Switzerland, gatherings of less than 1,000 people—under the assumption of strict safety concepts in place - were allowed again as of June 2020. The Swiss Societies for Infectious Diseases (SSI) and for Hospital Hygiene (SSHH) therefore decided to hold their annual congress, with an expected number of 300 participants, corresponding to an event with higher risk according to the CDC document [3]. Safety concepts of large gatherings have not been systematically evaluated [8]. In Austria, the Salzburg Festival took place in August 2020 with a total of 70,000 visitors for 90 performances over 30 days ^1^. One positive staff-member was reported, without any secondary transmission. To our knowledge, there is no data evaluating the safety of medical congresses during the COVID-19 pandemic. Furthermore, little is known about the perception of the risk and safety concept of congress attendees.

In this prospective cohort, we intended to evaluate the safety concept of the Joint Annual Meeting of the SSI and SSHH by documenting the number of COVID-19 cases among congress attendees after the event. We also aimed to assess risk factors for potential infections during the congress and to assess the risk perception of attendees.

## Methods

### Setting, Venue and Safety Concept

The congress took place in Geneva (Switzerland) between September 2nd and September 4th, 2020, at a time when the number of cases was rising again nationwide and after the first COVID-19 wave had been successfully contained. The Geneva region was particularly affected by the COVID-19 pandemic and showed one of the highest COVID-19 incidences in the country (65 cases/100,000 population in Geneva vs. 29 cases/100,000 population for Switzerland) during the week of the congress. The circulating strain at that time was the SARS-CoV-2 wild type strain ^5^. The congress took place at Palexpo, an exposition and congress centre offering 106,000 square meters of floor space in a single block hall. The Palexpo exhibition halls are among the highest in Europe (26.5 m high below the roof); the ventilation system consists in an open loop system ^2^. Only meetings that complied with the conditions imposed by the council of state were allowed to take place. Safety measures included universal masking (with surgical masks) in the exposition centre (except for the dining area); physical distancing during sessions (with a vacant seat between participants); hand hygiene upon admission to the congress (supervised by safety personnel); multiple dispensers of alcoholic hand rub within the congress area; personal data registration at lunch for contact tracing purposes; only seated catering and cancelling of official social events (Table 1). The pictograms used as part of the safety concept of the exposition centre are shown in Supplementary Figure S1. All congress participants received instructions on the necessary hygiene measures during the congress via email^3^.

**Table 1:** Safety concept of the congress venue.

Elements of safety concept
Obligatory mask at all times except while eating/drinking during breaks
Physical distancing 1.5 m
Cleaning
Regular disinfection of all surfaces with which participants have been in contact
Keeping doors open as much as possible
Sufficient and regular room ventilation
Regular refill of dispensers for soap, hand disinfectants, disposable towels and cleaning products
Regular cleaning and disinfection of toilets
Registration/congress environment
Payment in advance online or by credit card
Registration office staff works behind protective glass
No congress kits
Cloakroom on self-service basis
Exhibition measures
Sufficient distance between exhibition stands and maximum number of staff allowed simultaneously according to available space (2p/6m^2^; 3p/8 m^2^, 7p/15 m^2^, 11p/24 m^2^, 16p/36 m^2^, 18p/42 m^2^)
Regular disinfection of items displayed on the stand
Keeping minimum distance (1.5 m) to customers
Telephone number of staff available in case of contact tracing
Catering
Generally seated
Sufficient numbers of coffee stations to reduce waiting lines
Pastries, salads and desserts individually wrapped
Three different areas allowing a maximum of 100 people to be seated/catering area
Use of organic disposable material
For Lunch: a form is to be filled in by all seated person on each table and at each lunch
Dish of the day served by staff, buffets protected by Plexiglas
Water and juice served by an waiter
No networking dinners except for speaker’s dinner
Conference rooms, posterwalks
Every second chair used
Speakers bring their presentation on a USB key
No touching of microphones by the speaker
Regular disinfection of lectern computer and microphones
Authors presenting their poster during the posterwalk must also wear a mask
Personal data
Data of participants, sponsor/exhibitor delegate, organizations/congress employees stored for at least 14days after the event, treated with utmost confidentiality and deleted afterwards
In case of infection precautionary measures such as quarantine/isolation will be imposed on certain persons

Safety evaluation of a medical congress held during the COVID-19 pandemic–a prospective cohort, Switzerland, 2020.

### Recruitment and Study Procedures

All congress attendees (including participants, industry representatives and congress staff) >16 years old were informed by email before the congress about the study and were invited to participate in this prospective cohort. For study inclusion, oral informed consent was obtained from participants (approved by the local ethics committee).

Capillary dried blood spots (DBS) from a finger-prick applied on a filter paper card were collected from participants by trained personnel during the congress. Participants received an additional DBS collection kit labelled with their unique study ID and were instructed on how to obtain the follow-up blood sample. The kit contained an instruction sheet (English, German or French), alcohol swabs, auto-retractable safety single-use lancet, a filter paper card, plasters and a return label. Participants were requested to collect finger-prick DBS 4 weeks (±7 days) after the congress and to send the filter paper to the study centre by postal mail. DBS cards were kept at −80°C until analysed.

Four weeks after the congress, an anonymized online questionnaire was sent to all congress attendees. Study participants were asked to enter their study ID in order to match the questionnaires with serology results. Congress attendees not included in the serologic investigation were also invited to fill in the questionnaire. Questions included the type of profession, working canton, canton of residence, the number of contacts with COVID-19 confirmed patients, adherence with protective measures before, during and after the congress (at the congress site but also off-site during private gatherings), adherence to safety measures of other congress attendees (i.e., peer rating), perceived risk of COVID-19 at the congress and the estimated impact of congress safety measures, use of public transportation, leisure activities before and after the congress. Also, participants were asked regarding symptoms compatible with COVID-19, and results of SARS-CoV-2 PCR swabs or serologies done before and after the congress.

### SARS-CoV-2 Serology From DBS

Before performing serology, filter paper cards (standard 903™ Protein Saver Cards, Eastern Business Forms, Greenville, United States) were punched and eluted. The methodology was previously developed and established in our research laboratory as part of another research project [9] All five circles were completely punched to 8 mm discs, incubated with 0.5 ml elution buffer for 180 min on a shaker at room temperature. Finally, serology from the eluent was performed by two chemiluminescence immunoassays (ECLIA, pan immunoglobulin with anti-nucleocapsid (N)- and anti-spike (S)-specificity, Roche Diagnostics, Rotkreuz, Switzerland). The latter assay simultaneously detects IgG, IgM and IgA directed against the receptor binding domain (RBD) of the S1 subunit of the S protein of SARS-CoV-2 and has been evaluated by our group before [10] Samples were deemed positive if either assay returned a result above the manufacturer’s cut-off.

### Outcome and Data Analysis

The primary outcome was a composite of self-reported COVID-19 diagnosis (i.e., SARS-CoV-2 positive nasopharyngeal PCR) within 3 weeks or seroconversion within 4 weeks after the congress. The secondary outcome was self-reported occurrence of COVID-19 compatible symptoms within 2 weeks after the congress (i.e., testing criteria of the Federal Office of Public Health). Baseline characteristics were described using number and percentages for categorical, and mean and standard deviation (or median and interquartile range, as appropriate) for continuous variables. The proportion of participants with the primary or secondary outcome was calculated; positive cases including previous diagnosis of COVID-19 were described in detail. We used SPSS (IBM SPSS Statistics 20, Armonk, New York) for all statistical analyses.

## Results

**FIGURE 1:**
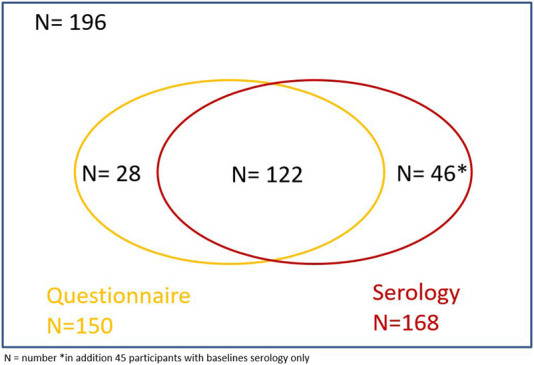
Venn diagram of congress attendees (n = 365), including number of participants who answered the questionnaire and who provided blood samples. Safety evaluation of a medical congress held during the COVID-19 pandemic–a prospective cohort, Switzerland, 2020.

A total of 365 individuals attended the congress, thereof 271 healthcare workers (HCW). Of these 365, 196 (54%) either answered the questionnaire (n = 150, including 17 without baseline serology) or provided baseline and follow-up blood samples (n = 168) (Figure 1). For 11 participants, the questionnaire could not be linked to the blood sample because of missing or incorrect ID. Questionnaire and blood samples could be matched for 122 participants.

Baseline characteristics of participants who answered the questionnaire, as well as risk exposures before, during and after the congress are summarised in Table 2. Among the 150 participants, 56% were physicians and 24% were infection control practitioners. The majority (58%) was female, 85% were between 30 and 60 years old, most of them (77%) lived in the German part of Switzerland, 23% in the French or Italian part. Regular contact with COVID-19 patients was reported by 50% of participants. Before the congress, 57 (38%) participants had a nasopharyngeal PCR for SARS-CoV-2, whereof 2 (3.5%) were positive. In the 3 weeks after the congress, eight participants reported having had a PCR for SARS-CoV-2 of which all were negative.

**Table 2:** Baseline characteristics and risk factors of study participants before/during and after the congress.

Baseline characteristics	All (n = 150)
Female, n (%)	87 (58)
Age, y (30–60)	124 (83)
Country of residence	
Switzerland, n (%)	140 (93)
French/italian part, n (%)	34 (23)
German part, n (%)	115 (77)
Profession	
Physician	84 (56)
Hospital hygiene expert	36 (24)
Other	30 (20)
Living with children	
>12 years, n (%)	39 (26)
≤12 years, n (%)	45 (30)
Contact with COVID-19 patients	
yes, <10, n (%)	37 (25)
yes, >10, n (%)	38 (25)
Risk exposure before congress	
Contact to covid infected persons 3 weeks before congress	
yes, overall, n (%)	32 (21)
yes, at work with patients, n (%)	27 (18)
Transport to congress	
public transport <1h, n (%)	13 (9)
public transport >1h, n (%)	110 (73)
Recreational activity	
none, n (%)	78 (52)
indoor/outdoor sports, n (%)	53 (35)
choir/music group, n (%)	9 (6)
Risk exposure during congress	
Accommodation	
At home, n (%)	41 (27)
Hotel single room	94 (64)
During congress attended	
Restaurant/Bar, n (%)	94 (63)
1–5 people met/evening, n (%)	50 (68)
5–10 people met/evening, n (%)	18(25)
Concert/Discothek/night club, n (%)	2 (1)
Risk exposure after congress	
yes, overall, n (%)	40 (27)
yes, at work with patients, n (%)	29 (19)
After congress attended	
Restaurant/Bar, n (%)	106 (71)
Concert/Theater, n (%)	22 (15)
Recreational activity	
none, n (%)	78 (52)
indoor/outdoor sports, n (%)	53 (35)
choir/music group, n (%)	9 (6)
Public transport used since congress	
never/sporadically, n (%)	75 (50)
during working days, n (%)	39 (26)
weekends, n (%)	12 (8)
during workings days/weekends, n (%)	24 (16)

Safety evaluation of a medical congress held during the COVID-19 pandemic–a prospective cohort, Switzerland, 2020. n, number.

Among 168 follow-up serologies, five (3%) were positive (Table 3). All five were already positive at baseline, resulting in zero seroconversions. Of the five seropositive cases, one did not provide a questionnaire, and two reported either a previous positive PCR and/or positive SARS-CoV-2 serology result. One participant reported a positive PCR before the congress, but had a negative serology both at baseline and at follow-up.

**Table 3:** Baseline characteristics, risk factors for study participants with positive serology or self-reported PCR/Serology.

	Baseline characteristics (sex/age)			Serology (baseline P1/follow-up P2)	Reported PCR/Serology	Risk factor
	Sex/age	Origin	Profession			
1	f/41–50 years	german speaking part of switzerland	Hospital pharmacist	pos/pos	not done/pos	pos house-hold contact
2	f/51–60 years	french speaking part of switzerland	Nurse	pos/pos	not done	pos house-hold contact
3	f/51–60 years	german speaking part of switzerland	Nurse	pos/pos	pos/pos	travelled outside switzerland
4	f/31–40 years	german speaking part of switzerland	Physician	pos/pos	not done/not done	contact with Sars-CoV-2 pos patient, travel outside Switzerland
5	f/31–40 years	german speaking part of switzerland	Physician	neg/neg	pos/pos	contact with Sars-CoV-2 pos patient, regular use of public transport
6	no info	no info	no info	pos/pos	no info	no info

Safety evaluation of a medical congress held during the COVID-19 pandemic–a prospective cohort, Switzerland, 2020. PCR, polymerase chain reaction; pos, positive; neg, negative; f, female.

**FIGURE 2:**
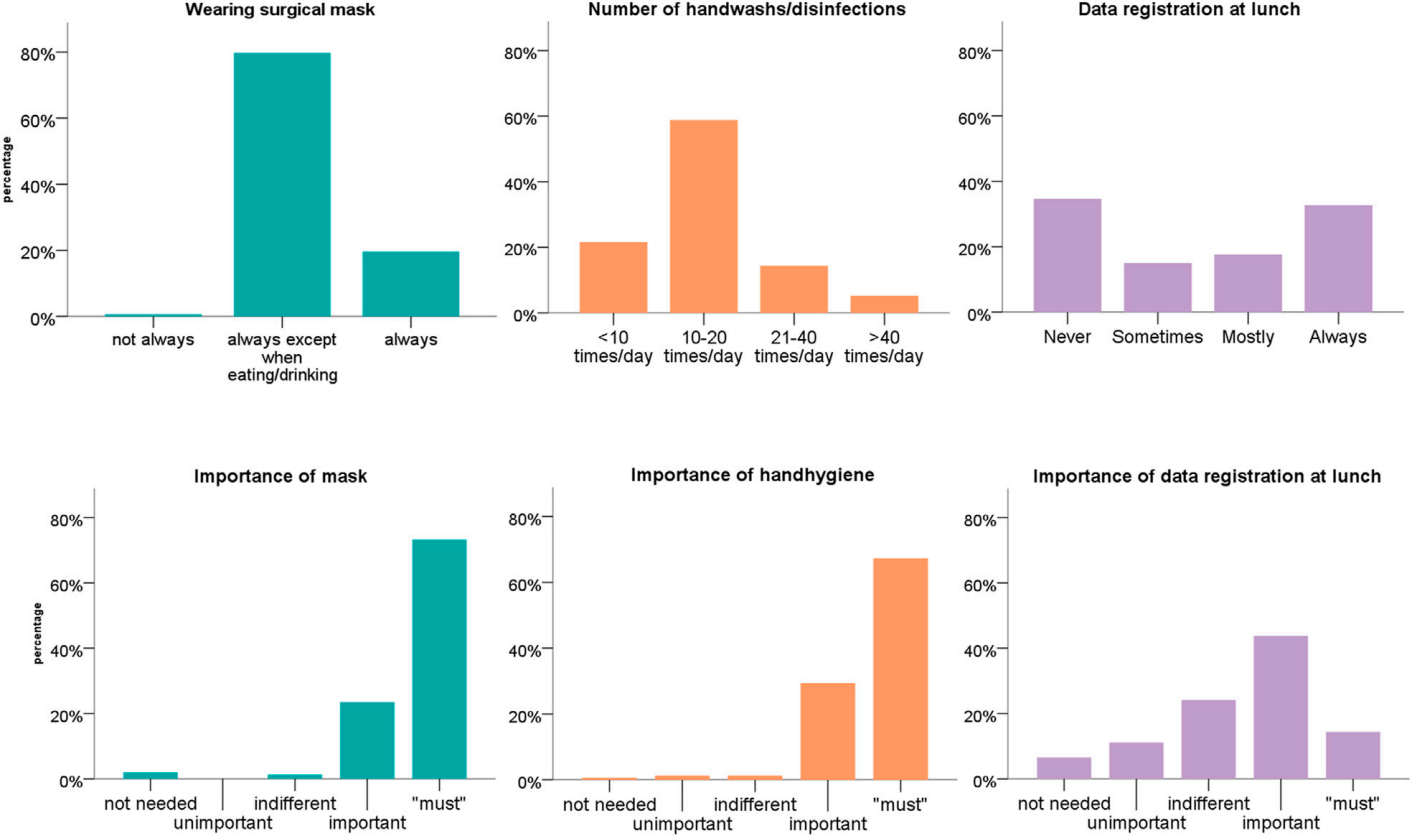
Self-reported adherence (upper row) and importance (lower row) of selected infection prevention measures of the safety concept. Safety evaluation of a medical congress held during the COVID-19 pandemic–a prospective cohort, Switzerland, 2020.

The safety concept was deemed appropriate by 92% of participants. Figure 2 shows the self-reported adherence of study participants with wearing a mask, hand hygiene, and data registration at lunch (upper row), as well as the perceived importance of the corresponding measures (lower row). Of note, 83% of respondents enjoyed the congress and preferred an on-site congress over a virtual one.

## Discussion

In this prospective cohort of unvaccinated attendees of an on-site medical congress held in a geographical area with low to moderately-high COVID-19 prevalence, we could not identify any SARS-CoV-2 cases or transmission events. At that time, the SARS-CoV-2 wild type was circulating in Switzerland ^5^. The findings underline the appropriateness of the safety concept, which was well accepted and followed by the participants. Strengths of the study are the dual approach to identify COVID-19 among participants using an online questionnaire and serology tests and the prospective design with a 4-week follow-up.

In our study, no SARS-CoV-2 transmission event was documented even though the congress was held in an area with moderately high prevalence, according the Federal Office of Public Health in Switzerland [9]. Also, congress attendees reported a certain risk exposure during the congress including use of public transportation [11], and spending time in restaurants and bars [12]. Data on the safety of mass gatherings during the COVID-19 pandemic are scarce. In Germany, the “Restart” study was performed investigating the safety and hygiene concept during a concert with several hundred participants. In addition to universal masking and rigorous hand hygiene, the safety measures included a smart working ventilation concept [13], which was considered as key measure [8] ^4^. The authors concluded that seated indoor events have a small effect on the spread of SARS-CoV-2 if a safety concept is in place. In our study, the different elements of the safety concept were perceived as differently important, which was also reflected by differences in adherence to these measures. Hand hygiene and wearing of masks, both basic infection prevention control measures, were perceived as essential and self-reported adherence was excellent. On the other hand, registration of personal data at lunch was not perceived as important and was therefore not universally followed.

During the COVID-19 pandemic many congresses are being cancelled to avoid gatherings of hundreds or thousands of attendees in a single venue [1]. These decisions might have significant consequences as medical education is being disrupted; also economic damage to scientific societies and industry partners has to be expected. Virtual congresses on digital platforms were established to overcome these problems. Many professional societies seem to have switched smoothly from physical to virtual congress [4], others even praised the advantage of easier accessibility for everyone, lower costs, and better environmental sustainability. However, virtual congresses have their downsides. Inspiration and emotions are less likely to be conveyed by online platforms; many formal and informal interactions based on human contact between congress participants from hospitals, societies and industry are limited. In line with this hypothesis, most study participants preferred the on-site version over a purely virtual congress. This finding has to be interpreted cautiously, as the people participating at the congress might not be representative for the entire community of Swiss ID and infection control professionals because of selection bias.

Of note, thirteen study participants reported symptoms compatible with COVID-19 within 3 weeks after the congress, but only half of them underwent PCR testing. This is surprising, as the FOPH recommends testing of all people, even those with minor symptoms. It seems that this recommendation is not universally followed, not even by people working in infectious diseases and infection control. However, since serology at follow up was negative in all eleven participants, an alternative cause for the reported symptoms is plausible.

### Limitations

Our study has weaknesses. First, most people besides congress staff participating in the congress were infectious disease physicians or hygiene experts, hence we assume adherence to hygiene rules were put consistently into practice. This might not be the case for attendees of other congresses. Second, almost half of congress attendees are not represented in our study. However, within the tightly connected ID community in Switzerland, potential undetected clustering of cases after the congress would have been noted by some of the society members. Third, the congress was held in September 2020, when SARS-CoV-2 vaccines were not yet available and where the wild type strain was circulating. Whether these findings can be applied to settings with vaccinated populations and with other prevailing variants such as the Omicron variant (B 1.1.529) - which has been shown to be considerably more infectious - remains unclear [1, 4]. Fourth, the accuracy of DBS in detecting SARS-CoV-2 antibodies has not been widely assessed [15]. However, study participants with previous positive PCR and/or positive serology were all seropositive in the DBS, except for one participant with positive PCR and serology in March. In this context it is important to note that almost 10% of patients with positive PCR results do not show specific SARS-CoV-2 antibodies or decline over time [16]. These results and the fact that the seroprevalence of 3% is perfectly in line with previous seroprevalence studies among Swiss HCW from the same time period strengthen our confidence in the DBS methodology [9].

### Conclusion

In summary, these results suggest that on-site medical congresses held in geographical areas with low to moderately-high COVID-19 prevalence (with the SARS-CoV-2 wild type mainly circulating within an unvaccinated population) can be safely held, provided that appropriate safety measures are implemented and meticulously followed. The safety concept used at this congress could serve as a model for future similar events. However, adjustments of safety measures might be necessary given that a large part of the population is immunised against SARS-CoV-2 and that the infectiousness of newer virus variants has increased in the meantime.

## Footnotes

1
https://www.salzburgerfestspiele.at/en/sf-2020

2
https://palexpo.ch/fr/sante-securite

3
https://www.meeting-com.ch/fileadmin/user_upload/programme_pdf/Divers_2020/SAFETY_CONCEPT_SSI-SSHH_2020_E.pdf

4
https://www.aerzteblatt.de/archiv/216565/Studie-RESTART-19-Konzerte-in-Zeiten-von-Corona

5
https://www.covid19.admin.ch/de/epidemiologic/virus-variants

## Supplementary Material

Supplementary Figure S1 Pictograms used at the congress venue as part of the safety concept. Safety evaluation of a medical congress held during the COVID-19 pandemic – a prospective cohort, Switzerland, 2020

## Abbreviations

DBS, dried blood spots; CDC, centers for disease control and prevention; COVID-19, coronavirus disease-2019; ECLIA, electrochemiluminescence-immunoassay; FOPH, federal office of public health; HCW, health care worker; ID, infectious diseases; PCR, polymerase chain reaction; SARS-CoV-2, severe acute respiratory syndrome coronavirus-2; SPSS, statistical package for the social sciences.
