# Supplementary figures and images for: Safety Evaluation of a Medical Congress Held During the COVID-19 Pandemic—A Prospective Cohort
Source: Int J Public Health. 2022 Feb 16;67:1604147. doi: 10.3389/ijph.2022.1604147 (PMC8889572; doi:10.3389/ijph.2022.1604147)

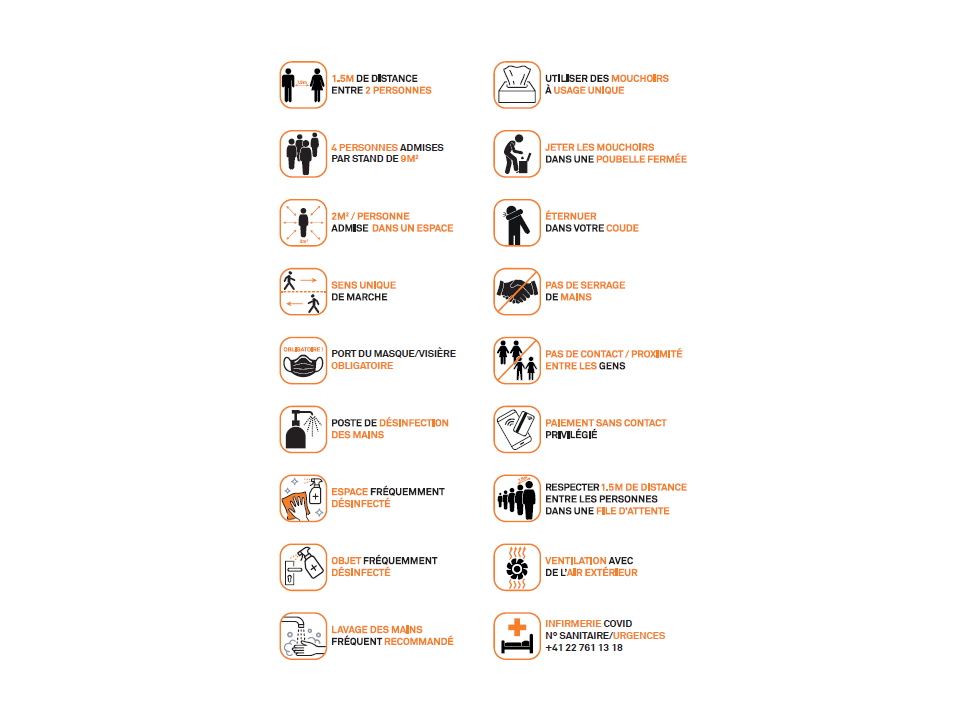

Supplement: Supplementary file 1 [file Image1.TIF]
